# Shifting focus from ideality to reality: a qualitative study on how quality of life is defined by premanifest and manifest Huntington’s disease gene expansion carriers

**DOI:** 10.1186/s13023-024-03461-x

**Published:** 2024-11-29

**Authors:** Pearl J. C. van Lonkhuizen, Anne-Wil Heemskerk, Leanne Slutter, Erik van Duijn, Susanne T. de Bot, Niels H. Chavannes, Eline Meijer, on behalf of the HEALTHE-RND consortium, Niko Vegt, Niels Chavannes, Anne- Wil Heemskerk, Susanne T. de Bot, Pearl J. C. van Lonkhuizen, Niko Vegt, Stephanie Feleus, Esther C Arendts, Amy Putman, G. Bernhard Landwehrmeyer, Alzbeta Mühlbäck, Wiebke Frank, Franziska Steck, Jiří Klempíř, Romama Konvalinková, Eva Bezuchová, Kristýna Dolečková, Olga Klempířová, Jan Roth, Olga Ulmanová, Ferdinando Squitieri, Sabrina Maffi, Eugenia Scaricamazza, Simone Migliore, Chiara Giorgio, Barbara D’Alessio, Melissa Casella, Jennifer Hoblyn, Muthukumaran Thangaramanujam, Tom Burke, Emer O’Malley, Stephen McKenna, Ian McKenna, Jeanette Thorpe, Anna Coffey, Ramona Moldovan, Peter Foley, Jacqueline Kerr

**Affiliations:** 1 Department of Public Health and Primary Care, Leiden University Medical Center, Postzone V0-P, PO Box 9600, 2300 RC Leiden, The Netherlands; 2 National eHealth Living Lab, Leiden University Medical Center, Leiden, The Netherlands; 3 Huntington Center Topaz Overduin, Katwijk, The Netherlands; 4 Department of Neurology, Leiden University Medical Center, Leiden, The Netherlands

**Keywords:** Huntington disease, Quality of life, Psychological well-being, Qualitative research, Neurodegenerative diseases

## Abstract

**Background:**

Understanding quality of life (QoL) is important in diseases for which there is no cure to date, such as Huntington’s disease (HD). A deeper level of understanding is, however, compromised by the lack of studies examining QoL from the perspectives of HD gene expansion carriers (HDGECs). Only a few qualitative studies on QoL in HD have been performed, yet none investigated how QoL is defined by HDGECs themselves.

**Objective:**

This qualitative study explores how premanifest and manifest HDGECs define their QoL.

**Methods:**

Online semi-structured interviews were conducted with 6 premanifest and 6 manifest HDGECs in the Netherlands. Qualitative content analysis was used to explore participants’ QoL definitions via inductive coding and the subsequent formulation of (sub)categories and (sub)themes.

**Results:**

Premanifest and manifest HDGECs had a different focus when defining QoL. Two subthemes were identified for premanifest HDGECs: *Thoughts about a meaningful life regardless of HD* and *Concerns about the future progression and impact of HD.* For manifest HDGECs, two other subthemes were identified: *Coming to terms with HD* and *Shifting perspectives due to the impact of HD.* One overall theme was generated, reflecting the difference and adaptive shift in focus between premanifest and manifest HDGECs: *Shifting focus from ideality to reality*.

**Conclusions:**

In providing optimal care, HDGECs should be considered as part of a complex, continuously changing environment, thereby taking into account their individual QoL experiences and tailoring care accordingly. HDGECs might benefit from forming helpful beliefs about future adaptability and resilience and developing adaptive coping strategies.

## Background

Definitions of quality of life (QoL) vary widely across the literature and its exact meaning is still a matter of ongoing debate [1–3]. In general, QoL can be considered a highly subjective experience of an individual’s functioning in life [3, 4]. QoL should be distinguished from health-related QoL (HRQoL) [5–7], as that the latter focusses solely on health-related aspects, whereas QoL includes more dimensions [1, 4, 8]. Common QoL dimensions measured across the literature include physical, psychological, and social functioning, and to a lesser extent dimensions such as spirituality, environment, cognition, and socio-economic factors [4, 8, 9]. The overall understanding of QoL is, however, complicated by the large variability in how QoL is being conceptualized and measured in the literature [8, 10].

Understanding QoL, and its underlying meaning for patients, is particularly important in diseases for which there is no cure to date, such as Huntington’s disease (HD). HD is a rare, autosomal dominant neurodegenerative disease resulting from a CAG repeat expansion in the *huntingtin* (HTT) gene [11, 12]. Children of an HD affected parent have a 50% chance of inheriting the gene expansion and developing HD [12]. The disease usually manifests between the fourth and fifth decade of life [13, 14], a time in which most HD gene expansion carriers (HDGECs) have an active family, work and social life. HD progressively affects motor, cognitive and neuropsychiatric functioning [11, 12, 14], resulting in an increased care need over time [12]. After the onset of manifest disease, as characterized by clinical motor changes [12–14], life expectancy ranges from 15 to 20 years [11, 14]. The manifest disease phase, is preceded by the premanifest phase in which (subtle) changes in motor, cognitive and/or neuropsychiatric functioning have not yet occurred (i.e., pre-symptomatic phase) or can already be present (i.e., prodromal phase) [15, 16].

Due to the serious consequences of HD in terms of personal, social and familial life [17, 18], the impact of HD on QoL is an important topic of study [7, 11, 12, 19], especially given that QoL is the main focus of treatment next to symptom management [11, 12]. Previous research has shown that QoL is experienced differently across the course of HD [7], with HDGECs in the manifest phase reporting lower scores on QoL measures compared to HDGECs in the premanifest phase [20] and people with other neurological conditions [21]. However, a recent systematic review on (HR)QoL in HDGECs suggests that a deeper level of understanding of QoL in HD is compromised by methodological shortcomings across studies, and the lack of a uniform definition of QoL to date [7].

Moreover, previous research on (HR)QoL in HD has a strong quantitative focus, whereas a qualitative approach examining the patients’ perspective might be more suitable given the subjective and temporary nature of QoL [7, 22]. Only three qualitative studies on QoL in HD have been performed, focusing on perceived QoL and QoL-related themes [23–25]. As there is no uniform definition of QoL [1–3, 26], and even less so for HD [7], it is essential to build on our understanding of QoL by exploring the definition of QoL from the perspective of HDGECs themselves [7, 22, 27]. The present qualitative study therefore aims to explore how premanifest and manifest HDGECs define their QoL.

## Methods

### Study design and participants

This qualitative study used a subset of data from a larger project (HEALTHE-RND HD-eHelp study) in which HDGECs, their partners and health care providers shared their experiences and needs with regard to QoL and eHealth, in order to co-develop a European eHealth application targeting QoL of HDGECs and their partners at home [28]. The current study used the data collected from Dutch HDGECs in individual interviews conducted between 2020 and 2021.

Participants were recruited via the Enroll-HD database held at the Department of Neurology of the Leiden University Medical Center by telephone via purposive sampling (based on gender and HD stage), provided that they agreed on being contacted for information on new HD studies. HDGECs were 18 years or older and still living at home. Participants had a genetically (in case of premanifest) or clinically (in case of manifest) confirmed diagnosis (i.e., based on the Unified Huntington’s Disease Rating Scale diagnostic confidence level of 4). Exclusion criteria were: having a serious psychiatric, neurological, sensory, or other comorbid disorder influencing participants’ judgement; the inability to give informed consent; the inability to attend all study sessions; having a partner participating in the study to prevent influencing each other’s judgement; and being part of the advisory panel of the study [28]. The in- and exclusion criteria guided the selection of participants for this study. Participants were selected by a physician of the Department of Neurology based on participants’ Enroll-HD records. Participants were approached until the target sample size of 12 was reached [28].

### Procedure

Interviews were guided by a semi-structured interview protocol consisting of questions on QoL, HD experiences and eHealth, and sensitizing assignments that participants filled in prior to the interview [28]. Sociodemographic information was self-reported and collected as part of the sensitizing assignments. The sensitizing assignments prompted participants to think about certain topics (e.g., current/future complaints, talking about HD, reflection on a day in life, and factors affecting QoL) prior to the interview [28]. This preparation could help participants to better express their experiences and needs during the interviews, including those that they were less aware of [29]. Participants’ responses guided the further course of the interviews.

Interviews were conducted digitally due to the COVID-19 restrictions at the time. All interviews were performed by a trained neuropsychologist (PL) and user-centered design expert, who were both present at each interview, yet alternated in conducting the interviews. All interviews were audio-recorded and transcribed intelligent verbatim while masking all personally identifiable information.

Detailed information on the study’s procedure and sensitizing assignments has been reported elsewhere [28]. The consolidated criteria for reporting qualitative research (COREQ) were used in guiding the reporting of this study [30].

### Qualitative analysis

Qualitative content analysis [31, 32] was used to explore the QoL definitions given by HDGECs. This type of analysis is considered suitable for studies aimed at describing a particular phenomenon of which little is known [33], such as how QoL is defined in HD, which was the focus of this study. Participants’ responses to the question *“What comes to mind when you hear the word quality of life?”* or similar questions (e.g., “*How would you describe your quality of life?”*, *“What do you think of when thinking of quality of life?”, “What does quality of life mean to you?”*) were selected as the units of analysis. Relevant responses to subsequent probing questions were included in the analysis as well. All transcripts were read by the first (PL) and third author (LS) in order to become familiar with the data. Meaning units were then selected from participants’ QoL definitions and coded inductively by the third author. Meaning units were subsequently condensed while preserving the core meaning, and double coded by the first author, thereby complementing and refining the initial coding scheme. Codes with similar meanings were grouped into (sub)categories by the first author, reflecting the manifest content of the text. Resulting (sub)categories, with their corresponding condensed meaning units and codes, were discussed and refined with the second (AH) and last (EM) author. This was followed by formulating (sub)themes through a discussion of the underlying meaning of the categories (i.e., latent content). Resulting subthemes and categories were reported separately for premanifest and manifest HDGECs. An example of the analysis schedule with (condensed) meaning units, codes, (sub)categories, and (sub)themes for this study is displayed in Table 1.

**Table 1:** Example of content analysis schedule: from meaning units to (sub)themes

Meaning unit	Condensed meaning units	Codes	Subcategories	Categories	Subtheme	Theme
The QoL is that informal caregivers are not burdened	That informal caregivers are not burdened	Not wanting to burden caregivers	Burden to others in surrounding	Effect future complaints on self/others	Concerns about future progression and impact of HD	Shifting focus from ideality to reality
Nevertheless, it [participant’s functioning] can only deteriorate	It can only deteriorate	It can only deteriorate	Progressive nature of HD	Perception of disease	Coming to terms with HD	Shifting focus from ideality to reality

*HD* Huntington’s disease

To ensure inclusion of different perspectives in data analysis, researchers with different backgrounds and expertise were involved (PL: neuropsychologist; AH: senior researcher with expertise in HD; LS: medical psychologist; EM: health psychologist with expertise in qualitative research). Moreover, we ensured trustworthiness of findings by reflecting on the concepts of *credibility* (i.e., validity of findings), *dependability* (i.e., reliability of findings over time), *transferability* (i.e., degree to which findings are applicable to other groups/settings), and *confirmability* (i.e., objectivity of findings) [31, 34, 35]. We included participants with a range of disease phases, age and gender, and described the (larger) context of the study (credibility and transferability); provided a clear description of the analysis process including an example of the analysis schedule and the display of quotations (credibility, transferability, and confirmability); involved multiple researchers with different backgrounds during analysis (credibility, confirmability); took preunderstandings into account (credibility); had continuous and open discussion about findings (credibility and dependability); and kept track of coding and relabeling decisions (dependability).

Transcripts were analyzed using ATLAS.ti version 23. Quotes corresponding to QoL definitions provided by participants were translated from Dutch to English by the first author, while keeping close to the original wording, grammar, and sentence structure used by participants. Translated quotes were subsequently checked and adjusted after review by the last author and a bilingual speaker (i.e., Dutch-British).

## Results

### Participants’ characteristics

Nineteen individuals were contacted to participate in this study, of whom 12 agreed and 7 declined. Reasons for declining included being too busy at the moment (n = 2); already participating in other HD research (n = 2); lack of response due to not answering the phone (n = 2); or because the study was too complicated/overwhelming at the time (n = 1). Of the 12 participants that agreed to participate, two dropped out prior to the start of the interviews as they were too busy at that moment (n = 1) or had difficulty with filling in the assignments as they did not experience HD to be a big part of life yet (n = 1). Subsequently, two additional participants were recruited.

In total, 12 HDGECs (6 premanifest, 6 manifest) participated in the interviews. Mean age of the included sample at time of the interview was 53 years, with premanifest HDGECs having a mean age of 46 years and manifest HDGECs of 59 years. Female to male ratio was 1:1 for both groups. Interview duration ranged between 1 h and 3 min to 1 h and 47 min. Other relevant sociodemographic information is displayed in Table 2.

**Table 2:** Sociodemographic characteristics of study sample at time of interview

	Premanifest HDGECs (N = 6)	Manifest HDGECs (N = 6)
Age (mean; range)	46; 31–56	59; 44–68
Gender (n (%))		
Male	3 (50)	3 (50)
Female	3 (50)	3 (50)
Living situation (n (%))		
Together with partner	5 (83)	4 (67)
Alone	1 (17)	2 (33)
Employment situation (n (%))		
Fulltime	4 (67)	0 (0)
Parttime	0 (0)	2 (33)
Retired	0 (0)	1 (17)
Unemployment benefit	1 (17)	1 (17)
Invalidity benefit	1 (17)	2 (33)
Time since genetic test in years (median; range)	8; 1–18	6; 1–14

*HDGECs* Huntington’s disease gene expansion carriers, *N* number of participants

### HDGECs’ definition of QoL

All participants provided a definition of QoL (see Table 3). The term QoL was understood by all participants, with none asking for clarification of its meaning. One premanifest HDGEC (P6) indicated that the question about QoL was quite difficult to answer but did not specify why, and nevertheless continued with providing a definition.

**Table 3:** Quotes of quality of life definitions provided by participants

Participant	Gender	QoL definition
*Premanifest HDGECs*		
1	Male	Well, basically I think [poor connection] about being healthy, being free of complaints and being able to deal with complaints. But in addition, I mostly think, the main problem with Huntington, is social well-being. And I think that is one of the issues. And for myself, for myself I’m a little less afraid of the future, but QoL is also the pressure you put on others. On informal caregivers, on those you live with. I also see that as part of QoL. Also going through life smoothly […] The QoL is that informal caregivers are not burdened, but that you can just live together as a family in that way. That is QoL. On the other hand, just thinking about it in a healthy way, but the social aspect is mainly… Now I have quite a few social contacts and things like that. That is an important thing that I am afraid to lose, certainly in relation to Huntington
2	Male	With QoL, it comes to my mind that you are not too limited by HD, so to speak. QoL is of course somewhat broader, because you can look at QoL more generally and then the question is: what is the QoL? So I did view it that way, in the context of Huntington, so to say, and what is QoL? And in what way is the quality lower? At this time, for me, I am satisfied and happy with the QoL now. Look, I would like to have more money because that would perhaps make the quality better, but not in a way that I have severe limitations that affect my QoL. So if I see that [QoL] with my mother, for example, she is very difficult to understand, she moves quite a lot. The moment you want to ask something and she wants to say something, she always starts to talk, then she starts grumbling, because she finds it hard to express herself. Look, then I say, your QoL… Other than that, it’s still fine. But those are then difficult things of QoL. I see that as lower quality […] Then it [QoL] becomes lower, or more limited. What I am afraid of, and which reduces QoL, is that instead of a pleasant family man, you become a distant grumpy, snappy man. I would find that… Then I would be sad about my QoL […] I would not like that. That [behavioral change] would be a violation of my quality, yes. […] The fact that you become less mobile also reduces QoL somewhat, but I would mind that less than the other thing [becoming a distant grumpy man]. I would have more problems with that
3	Male	QoL. Look, of course you hope not to become disabled or something. Look, I of course, a few years ago, I could no longer walk [for reasons other than HD]. Then you become dependent on your wheelchair because of my back. And then I’ve had that [description of treatment method] afterwards and I’m glad I can walk again now. And then of course you’re happy that you actually have, in my opinion, a good QoL, but I don’t really want to attribute that to Huntington. So that’s why I sometimes find some things quite difficult, then I think yeah, do I already have to think about Huntington? No, I don’t really want that. And then when I look at my sister, she feels incredibly down, and she’s just put under a lot of medication [in Dutch: kept stiff] and she then goes to [Huntington center] three times a week for a couple of hours because she can’t be alone. But this morning I was there again. I think what the hell is that kid doing in [Huntington center], because she’s not doing anything there. And she’s not getting any better either. And is only kept stiff with medication
4	Female	Yeah, that could be very broad, of course. It could be that you can still do everything and that your social circle is large. That you still earn your own income, which gives you satisfaction. But as the disease shows itself more and more, becomes more evident, I have also seen that in my father and my brother, both of them have Huntington. Then you start to appreciate different things. So you keep pushing those limits again and again. Focus on what you can achieve. What’s important, or what you can still do. But right now, I do indeed hang out with friends, go places, organize things, yes doing things with family. Yes, that is just very valuable and for me that is also, that is also part of QoL
5	Female	Well, at least that you are happy, and yes, of course that can be in many ways. At work, in your own private life. But it seems to me that the most important thing is that you are happy, depending on how you can realize that for yourself
6	Female	That you can shape your own life the way you want, that you are not dependent, yes how do I say that, that you can live independently, that you can allocate your own time, that you are free, to say so, to do whatever you want. Yeah […] Doing things, but I don’t know, what do you think about QoL, that is quite a difficult question […] Yes, those things that I have written down there, they are also part of it for me, in addition to the other things, that you just do your daily things, that you are having a good time. That you are not limited by your body, but also not by other people
*Manifest HDGECs*		
7	Female	Yes, quite good. I don’t really feel that it [HD] affects me yet. And I for example have my bicycle via the WMO [Dutch social support act], I have a special tricycle and other than that, everything runs here. Yeah, I’m still in the early stages of movement and stuff, I think. Yes, if I compare myself to my uncles and my grandmother and my… yeah, yeah
8	Female	I don’t know what exactly I wrote down anymore. For day and night rhythm, I wrote that gives me structure in my life. That’s the first element [in the workbook]. And the second thing is that I like to communicate with people around me about my disease, and also tell them how my disease is doing. So that I’m very open about it and therefore don’t hesitate if I’m just not doing alright, or that I experience more symptoms, or that it is sometimes temporary or that you… yes, I just like to share that with others. In any case, you are venting it for yourself, I experience that myself. And then they can also anticipate better, otherwise they don’t know how to anticipate. If you keep saying it’s going well, when it’s not going well, right, if you sometimes feel overstimulated by certain… and therefore also have a bit of mood swings. And that is not a pleasant experience for myself, nor for my partner. I’m not afraid to talk about that
9	Male	I did relate it [the question about QoL] to my Huntington past, my Huntington name, because I do have a problem myself. My wife passed away almost a year ago. Of course, that strongly influenced my life, my QoL. That is also the reason that I still continued to work, just to have some distraction indeed, some structure, which has all changed again because of the corona thing, because I have to work from home now. Nevertheless, for Huntington I just want to keep up to date with developments, and that support that can be provided which will be very needed in the years to come, when things can only get worse. Look, now it’s not yet so bad. I’m still pretty good, especially physically it is not too bad for me. A few years ago, I was worse. But I took part in a drug trial once, and it helped me. Nevertheless, it can only deteriorate. And then I think that contact with peers and indeed medical support, medical information, is useful
10	Male	Yeah, because of the disease, I just think seize the day. I know I’ll [unintelligible] and stay mobile, yes. So that’s why, because of my disease and now you are limited with everything. So every day that I wake up, I’m happy. That’s why
11	Male	Yes, in short, I mainly think about… yeah, happiness, isn’t it? That you can be happy with yourself, how you live with your [social] environment, well, let’s just say, especially your direct environment. It used to be mainly work and wife, children and now it is mainly children and grandchildren nowadays. That is actually a new happiness, isn’t it, if you can be satisfied with that, then I already have a lot of QoL. And in itself I don’t even think about… Because certainly if I, as a Huntington patient, think of well, I have to be completely healthy or something, you know? That’s not even necessary, because a lot of those kind of things, you can make that work. If you are no longer able to walk, well then, you may have to use a walker or a wheelchair. Nowadays there are quite a few possibilities to overcome all that and so I think a lot about how the children and grandchildren are doing, and my relationship with them. That is the most important thing in my life. So
12	Female	Yes, everything actually. Movement. But also being able to do everything at home and household, actually everything. With friends

*HDGECs* Huntington’s disease gene expansion carriers, *HD* Huntington’s disease, *QoL* Quality of life

**Fig. 1:**
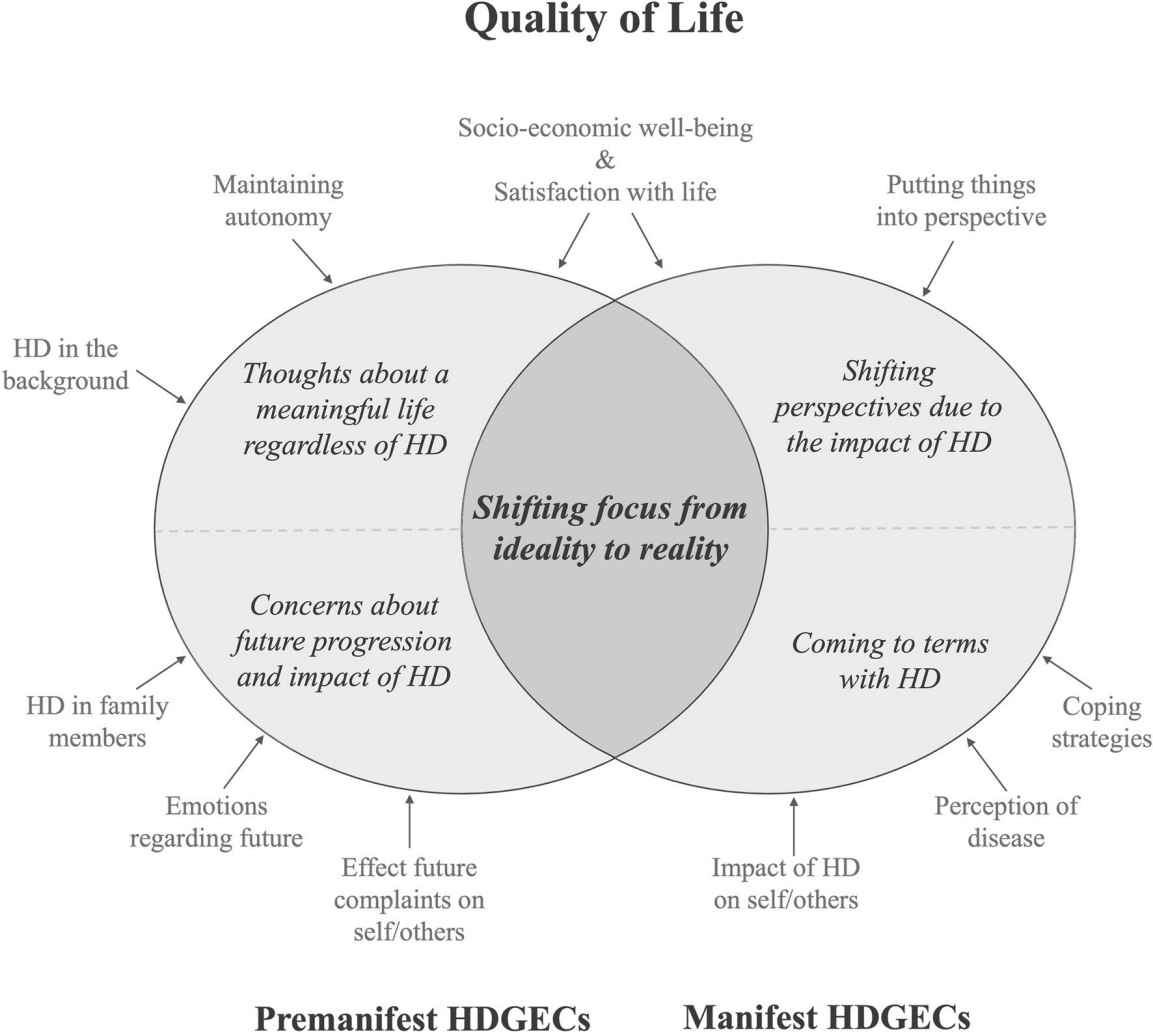
(Sub)themes and corresponding categories identified from quality of life definitions in premanifest and manifest HDGECs. HD: Huntington’s disease

One main theme was identified from the QoL definitions of all participants: *Shifting focus from ideality to reality*. This theme subsumed four subthemes, derived from 11 different categories (see Fig. 1). Two of the subthemes were identified among premanifest HDGECs: (1) *Thoughts about a meaningful life regardless of HD*, which describes the categories of socio-economic well-being, maintaining autonomy, satisfaction with life, and HD in the background; and (2) *Concerns about the future progression and impact of HD*, which involves references of participants to HD in family members, emotions with regard to the future, and effects of future complaints on self and others. The other two subthemes were identified among manifest HDGECs: (3) *Coming to terms with HD*, which describes the impact of HD on self and others, the perception of the disease, and coping strategies; and (4) *Shifting perspectives due to the impact of HD*, which illustrates putting things into perspective, socio-economic well-being and satisfaction with life. The main theme is illustrated by discussion of the underlying subthemes and categories per group below.

#### Premanifest HDGECs’ definition of QoL

Five premanifest HDGECs provided a comprehensive definition of QoL, whereas the definition of one participant (P5) was very concise (see Table 3). One participant (P2) incorporated an evaluation of QoL in his definition and stated that he was satisfied with his current QoL and did not experience limitations of HD affecting his QoL. None of the other participants included such an evaluation in their QoL definitions, yet one participant (P3) mentioned the impact of a former comorbid health disorder on his QoL.

##### Subtheme 1: Thoughts about a meaningful life regardless of HD

When defining QoL, all premanifest HDGECs expressed thoughts about a meaningful life regardless of the reality of HD.

A meaningful life was interpreted in different ways, and included factors related to socio-economic well-being, maintaining autonomy and satisfaction with life. Factors related to socio-economic well-being in relation to QoL were mentioned by more than half of all premanifest participants and discussed evenly among female and male participants. Some participants talked about the importance of economic factors, such as being able to generate one’s own income and getting satisfaction from work. One participant indicated that having more financial resources would improve his QoL. Others focused more on factors related to social well-being in their definition of QoL, including undertaking social activities and having social relationships:

*“But right now, I do indeed hang out with friends, go places, organize things, yes doing things with family. Yes, that is just very valuable and for me that is also, that is also part of QoL.”* (P4)

In addition to socio-economic well-being, which was the only aspect mentioned by (two) male participants, female participants also emphasized other aspects of a meaningful life. For instance, two female participants (P4 and P6) emphasized the importance of maintaining autonomy in relation to QoL. Multiple aspects were raised, including self-management and being able to function independently:

*“That you can shape your own life the way you want, that you are not dependent, yes how do I say that, that you can live independently, that you can allocate your own time, that you are free, to say so, to do whatever you want.”* (P6)

Besides the strong focus on maintaining autonomy, this participant also expressed that QoL refers to having a good time. Satisfaction with life was also raised by another female participant (P5) who focused solely on happiness in her definition of QoL and did not include any other factors:

*“But it seems to me that the most important thing is that you are happy, depending on how you can realize that for yourself.”* (P5)

She described the importance of being happy both at work and in personal life.

While discussing these constitutes of a meaningful life, all premanifest participants did not include their reality with HD when defining QoL. Half of the premanifest participants mentioned that QoL included being healthy, being free of complaints and not being (too) limited by HD, thereby viewing an ideal life as being free of limitations posed by HD. Another participant (P3), with a former comorbid health condition, was a bit hesitant about the cause of his complaints, which he did not want to attribute to HD:

*“And then [after being able to walk again following treatment of a comorbid condition] of course you’re happy that you actually have, in my opinion, a good QoL, but I don’t really want to attribute that to Huntington. So that’s why I sometimes find some things quite difficult, then I think yeah, do I already have to think about Huntington? No, I don’t really want that.”* (P3)

Two participants, both female, solely focused on the description of a meaningful life without including any references to being symptom free or other limitations posed by HD:

*“[QoL means that] well, at least that you are happy, and yes, of course that can be in many ways.”* (P5)

While focusing on an ideal, meaningful life, premanifest participants disengaged from present HD-related thoughts, viewing it as a concern for the future, as illustrated in the next theme.

##### Subtheme 2: Concerns about the future progression and impact of HD

The majority of participants expressed concerns about future deterioration inherent to the progression of HD, as well as the impact of the disease on themselves and others. This subtheme was identified amongst all three male participants and one female participant.

Half of the participants acknowledged the progressive nature and impact of HD in their definition of QoL, as illustrated by the included references to affected family members. One participant briefly mentioned the affected family members (P4), whereas two participants (P2 and P3), both male, talked in more detail about the symptoms that family members experienced:

*“So if I see that [QoL] with my mother, for example, she is very difficult to understand, she moves quite a lot. The moment you want to ask something and she wants to say something, she always starts to talk, then she starts grumbling, because she finds it hard to express herself. […] Other than that, it’s still fine. But those are then difficult things of QoL. I see that as lower quality.”* (P2)

This participant describes the symptoms he has witnessed in his mother. He acknowledges that QoL would decrease when certain symptoms of HD start to manifest. The other participant also strongly expressed his opinion on the treatment his sister received:

*“I think what the hell is that kid doing in [Huntington center], because she’s not doing anything there. And she’s not getting any better either. And is only kept stiff with medication [Dutch phrase that indicates that participant’s sister is being put under a lot of medication].”* (P3)

Only male participants expressed emotions related to the future progression of the disease. For two of these participants, who also included detailed references to HD in family members, these emotions seemed to be related to what they have seen in family members. They talked about possible losses due to HD including fear of physical deterioration (P2 and P3), such as declining mobility or becoming handicapped. One participant expressed his fear of behavioral change (P2):

*“What I am afraid of, and which reduces QoL, is that instead of a pleasant family man, you become a distant grumpy, snappy man. I would find that... Then I would be sad about my QoL.”* (P2)

One other male participant stated that he was not that fearful of the future, as he tried to think about it in a ‘healthy way’. However, when talking about his current social relationships, he did mention the fear of losing social engagement:

*“Now I have quite a few social contacts and things like that. That is an important thing that I am afraid to lose, certainly in relation to Huntington.”* (P1)

This participant was also concerned with the future impact of HD on others. He directly related QoL to his social environment, by stating that he did not want to burden people in his environment once the disease progresses:

*“…but QoL is also the pressure you put on others. On informal caregivers, on those you live with. I also see that as part of QoL.”* (P1)

He described that for himself, it would be important to cope well with future complaints. Two other participants also talked about the impact of future complaints on themselves. One of these participants (P2) acknowledged that the future manifestation of HD, and especially changes in behavior, would reduce his QoL. When he compared the effects of future decline in mobility and changes in behavior, he concluded that behavioral change would be worse as it would negatively impact his QoL:

*“[…] I would not like that. That [behavioral change] would be a violation of my quality, yes. […] The fact that you become less mobile also reduces QoL somewhat, but I would mind that less than the other thing [becoming a distant grumpy man]. I would have more problems with that.*” (P2)

Another participant included references to affected family members when talking about the effects of the progression of HD. She described how perspectives will change once the disease manifests, as had happened in her relatives with HD:

*“But as the disease shows itself more and more, becomes more evident, I have also seen that in my father and my brother, both of them have Huntington. Then you start to appreciate different things.”* (P4)

#### Manifest HDGECs’ definition of QoL

Half of the manifest HDGECs provided a detailed definition of QoL, while the other half defined QoL more concisely (see Table 3). One participant (P7) included an evaluation of QoL in her definition and indicated that her current QoL was quite good. None of the other manifest HDGECs provided such an indication about their QoL, yet one participant (P9) mentioned that having become a widower has strongly impacted his QoL.

##### Subtheme 3: Coming to terms with HD

From their definitions of QoL, it was apparent that the vast majority of manifest HDGECs came to terms with HD as they discussed the impact of HD on themselves and others, their perceptions of the disease, and ways to cope with their HD experiences.

One participant (P8) acknowledged that her current experienced symptoms, including being overstimulated and having mood swings, had an impact on herself and her partner:

*“And that is not a pleasant experience for myself, nor for my partner.”* (P8)

References to how participants currently perceive their disease were included in the QoL definitions of more than half of manifest HDGECs. Some participants acknowledged the progressive nature of HD, yet specific disease perceptions also differed across participants. One of the participants (P10) mentioned that he was currently ‘limited with everything’ due to HD, whereas another participant (P7) indicated that she did not feel that HD has affected her yet:

*“I don’t really feel that it [HD] affects me yet. And I for example have my bicycle via the WMO [Dutch social support act], I have a special tricycle and other than that, everything runs here. Yeah, I’m still in the early stages of movement and stuff, I think.”* (P7)

Although she mentioned that she did not yet experience the impact of HD, she did describe how she is currently in the early stages of motor symptoms and that she needs a tricycle for cycling.

The use of a medical aid, like a tricycle, is one way of managing experienced complaints. A variety of other coping strategies were discussed by the majority of manifest HDGECs. For instance, one female participant emphasized the importance of talking about HD with others:

*“And the second thing is that I like to communicate with people around me about my disease, and also tell them how my disease is doing. So that I’m very open about it and therefore don’t hesitate if I’m just not doing alright, or that I experience more symptoms, or that it is sometimes temporary or that you… yes, I just like to share that with others. In any case, you are venting it for yourself, I experience that myself.”* (P8)

Being open about HD to others seemed a very important aspect of her life as her definition of QoL was primarily focused on talking about the disease. This helped her to vent her experiences and emotions in a way that enables her to cope with all that comes along with HD. She also mentioned the importance of HD awareness among people around here, such that they are well-informed of her current HD status and able to anticipate her needs.

Other coping strategies discussed by participants included adding structure to life (P8), being actively involved with the disease (e.g., participating in research, looking up information) (P9), and the use of medication or medical aids (P9 and 7). One participant (P11) acknowledged that there are many aids available for dealing with current or future complaints, such as a wheelchair or walking aid (see Table 3). Another participant (P9) emphasized the importance of aids, especially when the disease progresses:

*“Nevertheless, it can only deteriorate. And then I think that contact with peers and indeed medical support, medical information, is useful.”* (P9)

This participant acknowledged the progressive nature of HD and thought ahead about ways to deal with this progression when needed. At the same time, the (future) progression of disease allowed him to put his current complaints more into perspective, as discussed in the next subtheme.

##### Subtheme 4: Shifting perspectives due to the impact of HD

The majority of manifest HDGECs included references to shifting perspectives due to the impact of HD when defining their QoL. This subtheme was identified amongst all three male participants and in one female participant.

One participant put his current complaints into perspective, in light of his past and expected future symptoms:

*“Look, now it’s not yet so bad. I’m still pretty good, especially physically it is not too bad for me. A few years ago, I was worse. But I took part in a drug trial once, and it helped me. Nevertheless, it can only deteriorate.”* (P9)

He acknowledged that a worsening of functioning is to be expected in the future and that his functioning could only deteriorate from now on. Another participant (P7) did not feel that the use of a tricycle for biking is limiting her, and she seemed to focus on the things that she is still able to do. Another participant (P11) devoted most of his QoL definition to discussing more general changes in perspectives on life:

*“It [happiness] used to be mainly work and wife, children and now it is mainly children and grandchildren nowadays. That is actually a new happiness, isn’t it, if you can be satisfied with that, then I already have a lot of QoL. […] Because certainly if I, as a Huntington patient, think of well, I have to be completely healthy or something, you know? That’s not even necessary, because a lot of those kind of things, you can make that work. If you are no longer able to walk, well then, you may have to use a walker or a wheelchair. Nowadays there are quite a few possibilities to overcome all that and so I think a lot about how the children and grandchildren are doing, and my relationship with them. That is the most important thing in my life.”* (P11)

This participant talked about finding a ‘new happiness’, which for him included shifting the focus from work, marriage and being completely healthy to his children and grandchildren. Their well-being and his relationship to them were now his main focus as the disease was progressing.

This participant also related satisfaction with this new happiness to a good QoL. QoL for him included being happy with oneself and the people in his environment. Factors related to social(-economical) well-being were also mentioned by others. Another male participant (P10) focused on being able to enjoy life as part of his QoL definition:

*“Yeah, because of the disease, I just think seize the day. […] So every day that I wake up, I’m happy.”* (P10)

Both subthemes were identified from the QoL definitions of all manifest HDGECs, except that of one participant (P12). Her definition consisted of fragmented sentences which were difficult to interpret:

*“Yes, everything actually. Movement. But also being able to do everything at home and household, actually everything. With friends*.” (P12)

Her definition seems to be more fitting within the subthemes found for premanifest HDGECs, as she solely referred to maintaining autonomy and having social relationships. This participant was just recently diagnosed with manifest HD (1 year prior to the interview date) after a prior misdiagnosis of dementia.

### Differences and overlap between premanifest and manifest HDGECs

Regarding the QoL definitions provided by all participants, premanifest HDGECs seemed to provide a more detailed definition of QoL (5 out of 6 participants). Half of manifest HDGECs provided a detailed definition, whereas the other half defined QoL more concisely. Some of these definitions were slightly fragmented (P10 and P12) and somewhat incoherent (P8 and P9).

Moreover, different subthemes were identified for both groups. It seems that the premanifest participants focused more on what an ideal, meaningful life should look like regardless of the reality of HD, whereas manifest participants focused more on living and dealing with the impact of HD in the present moment. In addition, premanifest HDGECs redirected their HD-related concerns to the future, whereas manifest HDGECs expressed how the current progression and impact of HD allowed them to put things more into perspective.

This difference in focus between groups was also apparent in the underlying categories of each subtheme. Premanifest HDGECs focused more on factors constituting a meaningful life, such as socio-economic well-being and maintaining autonomy. They did talk about concerns with regard to the impact of the disease and symptoms, but these were all related to the future and not to the present. Manifest HDGECs, however, focused on how they currently experienced the disease, how they coped with these experiences, and how that changed their perspectives; topics that were not raised amongst premanifest HDGECs. Furthermore, the density of subthemes was higher for premanifest than for manifest HDGECs, indicating that premanifest participants discussed more topics when defining their QoL as opposed to manifest HDGECs. With regard to similarities between the groups, two categories were frequently mentioned by both premanifest and manifest HDGECs, i.e., socio-economic well-being and satisfaction with life. Both groups considered social relationships, happiness and enjoyment important constitutes of QoL.

## Discussion

The present study was the first to explore how quality of life (QoL) was defined in HD by including the perspectives of both premanifest and manifest HD gene expansion carriers (HDGECs). Premanifest and manifest HDGECs had a different focus when defining QoL, as illustrated by the different subthemes identified in each group. For premanifest HDGECs, two subthemes predominated their definitions of QoL: *Thoughts about a meaningful life regardless of HD* and *Concerns about the future progression and impact of HD*. Two subthemes were also identified for manifest HDGECs: *Coming to terms with HD* and *Shifting perspectives due to the impact of HD*. One overall theme was generated from the QoL definitions of all participants, reflecting this difference and shift in focus between premanifest and manifest HDGECs when defining QoL: *Shifting focus from ideality to reality*.

This overall theme was reflected as a whole in the QoL definition of one premanifest participant (P4) who first described an ideal and meaningful life for herself, and, at the same time, acknowledged the shift in focus she had seen in manifest family members.: *“[…] Then you start to appreciate different things. So you keep pushing those limits again and again. Focus on what you can achieve. What’s important, or what you can still do”*. The difference and shift in focus make sense, given the relatively distinct situations that premanifest and manifest HDGECs are in. Bloch, Adam [36] suggested five different stages of psychological response to the genetic and clinical diagnosis of HD, including warning, incipient, breakthrough, diagnosis, and adjustment. At first, individuals become aware of their genetic risk (warning) and eventually their own risk/genetic status (incipient). They slowly begin to experience symptoms (breakthrough) that ultimately result in the clinical confirmation of HD (diagnosis) and learning to cope with its consequences (adjustment) [36]. Due to the progressive and complex nature of HD, HDGECs continue to adapt to different situations across the course of their disease, as reflected in the differences in focus found in the present study.

Premanifest HDGECs in this study mainly focused on the constitutes of a meaningful life and discussed their ideal situation, which included being healthy, free of complaints and not limited by HD. This is not surprising given that the majority of premanifest participants did not report any current symptoms, except for one participant reporting sleep difficulties. At the same time, premanifest participants did acknowledge the future impact of HD and expressed concerns about future aspects they fear to lose in relation to HD. These findings are in line with previous studies showing that premanifest HDGECs seem to embrace their present and focus on a meaningful life [27, 37] by disengaging from current HD-related thoughts [37]. As symptoms are often subtle or have not occurred yet, individuals in the premanifest phase might easily redirect any HD-related concerns to the future and distance themselves from their own deeper emotional experience which might prevent them from being overtaken by fears [36]. This might allow them to hold on to their ideal situation and focus on meaningful aspects they consider important to maintain once the disease progresses.

Manifest HDGECs, on the other hand, seem to focus on their actual reality and what they are still able to do, given their current complaints. This shift in focus from the ideal to the actual situation could be inherent to the process of accommodating to a disease [38]. When faced with changes in health, individuals might change their values and standards to adapt to these new circumstances, a phenomenon known as response shift [38, 39]. This could have resulted in a different (redefined) conceptualization of QoL in manifest HDGECs, as opposed to premanifest HDGECs. As HD slowly progresses, the disease takes on a more prominent role in the lives of manifest HDGECs [36, 40]. This was also the case in the manifest participants included in our sample, as all self-reported to experience HD-related symptoms. In response to these deteriorations in health, manifest HDGECs might adapt to the progressive nature of HD by changing their values and standards. In our sample, manifest participants focused on ways to deal with these changes, and at the same time reappraised certain aspects of their lives, such as by putting their current complaints into perspective and finding ‘new happiness’. Premanifest HDGECs, however, did not seem to recognize this future capacity to adapt to deteriorations in health, a phenomenon described as inaccurate affective forecasting [41, 42]. Premanifest participants mainly focused on aspects they fear to lose rather than what will remain positive, thereby overlooking that these initial beliefs might be reconsidered and evaluated differently once the disease progresses. This resembles the so-called ‘disability paradox’, in which health status or QoL of patients is often underestimated by individuals that do not have the disease (yet) [43, 44]. As a result, premanifest HDGECs may not take their own future adaptability and resilience into account.

It should be noted that psychological adjustment is a dynamic, rather than a linear process [36, 45]. The suggested shift in focus reported here should therefore not be interpreted as inherent to transitioning from the premanifest to the manifest phase. In the current study, premanifest HDGECs already seem to balance between their ideal situation and their future reality by acknowledging the inevitable consequences of HD that await them, indicating the continuous adaptation throughout the course of the disease. This corresponds with previous research showing that individuals in the premanifest phase adjust well to their positive genetic test on the long-term [46] and, as a result, already reappraise the meaning of the present and alter their future expectations [27, 37, 47]. This suggests that response shift might transcend the mere objective deteriorations in health and can include the notion of changes expected in the future. Moreover, although general subthemes were identified from the QoL definitions of all participants, it should be acknowledged that psychological adjustment is a process that can vary widely across individuals and over time [45]. Some participants emphasized different aspects when defining QoL. This could be due to previous (disease) experiences, such as other comorbid diseases or becoming a widower, as was the case for two participants in this study. For one manifest participant, the QoL definition seemed more fitting within the subthemes found for premanifest HDGECs. This participant was previously misdiagnosed with a form of dementia. She explicitly stated that she was happier with her HD diagnosis than the former dementia diagnosis, as she believed her life expectancy to be more favorable with HD. The relatively short time period between the genetic test and the interview (i.e., 1 year) might have resulted in little time to adapt to the positive test result, let alone to both the test result and the manifest HD diagnosis. This could explain why subthemes identified among premanifest participants were reflected in her definition of QoL as she just seemed to learn about HD and her diagnosis. In addition, gender and age could also play a role in how individuals perceive QoL and adjust to (expected) changes in health [48–51]. In the present study, mainly males discussed concerns related to the future (in case of premanifest) and shifted their perspectives (in case of manifest). To avoid loss of potentially relevant information, we highlighted participants’ gender in findings where gender seemed relevant, however, we acknowledge that with the small sample size these gender-related differences should be interpreted with caution. Previous studies did not find a relationship between (health-related) QoL and gender in HD, and only mixed results were found for its relationship with age [7]. Moreover, a recent study suggest that reappraisal is not limited to older people, as young individuals in the premanifest phase already started to (positively) change their attitude and approaches to life [47]. We therefore consider it less likely that the shift in focus reported in the current study was inherent to older age in the manifest group.

This also touches upon the subjective nature of QoL and the importance of asking individuals about their own experiences [7, 22]. By doing so, qualitative studies allow for an enriched understanding of QoL as they reflect themes HDGECs consider important to discuss, such as interpersonal relationships, coping, HD manifestations in others [25], changes in identity, disease progression, uncertainty, and loss of control [24]. These topics are difficult to integrate with findings from quantitative studies on QoL in HD [7], as they are often not addressed in well-known QoL measures [4, 8, 9, 52] and these measures might therefore not truly reflect HDGECs’ QoL. As there is no uniform definition of QoL to date [1–3, 26], the perspectives of HDGECs on how they define QoL should therefore be taken into account. Our findings suggest that a uniform definition of QoL in HD is likely not desired given the differences in focus found between premanifest and manifest HDGECs when defining QoL. If one definition is to be considered, for instance for its convenience in research, it should be flexible in nature and leave room for the highly subjective nature of QoL. The definition of QoL provided by the World Health Organization (WHO) provides a good basis for use in HD: “individuals’ perception of their position in life in the context of the culture and value systems in which they live and in relation to their goals, expectations, standards and concerns” (p. 1405) [4]. Based on our findings, we suggest some slight additions to this definition for its use in HD: (1) to add both the *current* and *future* position in life, as HDGECs in our study related their QoL not only to the present but also to their expected future position, this was especially the case for premanifest participants; (2) to substitute *in relation to* with *continuing adaptations to* goals, expectations, standards and concerns, as previous studies and the current study suggest that HDGECs seem to continuously adapt to (expected) changes in their health. Given that these additions are quite general in nature, we consider these changes also relevant for other diseases as well as the general population. Nevertheless, we recommend to always ask HDGECs about their own experiences when it comes to QoL, both in research and in practice.

Some shortcomings of this study should be acknowledged. First, we included participants who were willing and able to participate in the current study thereby excluding participants with serious comorbid conditions, which resulted in a relatively well-functioning and well-motivated sample. All participants in our sample seem to adjust adaptively to (changes in) their situation, however, this is not always the case, especially not in the later stages of HD (e.g., denial, unawareness [53, 54]). Our findings may therefore not be representative of HDGECs in the broader HD population, yet we aimed to include participants with different disease phases and gender, as variety in perspectives contributes to a richer understanding of a phenomena [31]. This allowed us to build a first understanding of how QoL is defined in HD. Although the sample size can be considered relatively small, this is not unusual given the rarity of the disease [55]. Moreover, the current sample size is considered appropriate for reaching data saturation in qualitative research [56] as well as for user testing eHealth prototypes in the larger project of which the current study was a part. In the larger project, data saturation was reached after 12 interviews [28]. As this larger project aimed at developing a European eHealth application targeting QoL of HDGECs and their partners, it is likely that participants discussed QoL only in relation to HD. It should be noted that QoL is a broad construct that includes more dimensions than health (status) alone [2, 4]. As the full interviews were not analyzed in detail due to large parts being related to eHealth, our understanding of how QoL is defined in HD only reflects the information participants shared during (parts of) the interview, whereas unshared information might have been equally relevant or important to their QoL. Both the first and third author familiarized themselves with the full transcripts prior to the analysis. To encourage participants to better express their experiences and needs during the interviews, sensitizing assignments were filled out, prior to the start of the interview. It is possible that the assignments could have biased how participants defined their QoL, however, we find that unlikely because the assignments did not focus on QoL definitions. Instead, they comprised open-ended questions related to the larger study’s topics, including the disease, home environment, social circles, eHealth and factors participants considered important for QoL. The assignments were drafted in a way that participants become more aware of experiences and needs that are in the back of their minds or of which they were less aware of [28]. No difficulties were reported with filling in the sensitizing assignments. Third, as the interviews were conducted via a secured videoconferencing tool due to COVID-19 pandemic at the time, participants with lower digital skills could have been less likely to participate. Nevertheless, digital skills were not reported as reason for non-participation and no major problems with connectivity occurred during the interviews. Another potential drawback of video-conferencing could be reduced willingness to talk openly about experiences due the lack of personal connection between the participant and interviewer. The data quality of interviews performed via video-conferencing tools is, however, shown to be comparable to that of in-person interviews [57–59]. Lastly, some bias due to the researchers’ preunderstandings might have been introduced while analyzing the results [31, 32], yet minimized by involving multiple researchers with different backgrounds in the analysis process. Although interpretation of the data is key in qualitative research, it should be noted that multiple meanings can be inferred from a single text [31] and therefore our analysis of the data should be considered as one possible interpretation. We ensured trustworthiness of findings by providing all the quotes used in the analysis and by including some key examples of how we progressed from the raw interview data to (sub)themes [32]. For a detailed description of how the concepts of trustworthiness have been ensured during this study, the methods section can be referenced.

Some strengths are worth mentioning as well. To our knowledge, this is the first study to explore how HDGECs define their QoL. We used both manifest and latent content analysis to provide a first in-depth look into the components of HDGECs’ QoL definitions, as well as its underlying meanings. This analysis method is considered suitable for exploring phenomena about which little is known [33]. Moreover, by including both premanifest and manifest HDGECs we were able to give insights into how QoL is defined throughout the course of the disease. As the current study was cross-sectional in design, allowing for a first snapshot of how QoL is defined in HD, longitudinal studies are needed to explore whether the shift in focus from ideality to reality is observed in individuals over time. Moreover, it may be relevant to consider (changes in) QoL in the context of gender, age, and other relevant sociodemographic characteristics that were not included in this study. Given the different situations premanifest and manifest HDGECs encounter, studies should focus on developing separate QoL questionnaires for premanifest and manifest HDGECs based on their perspectives. Moreover, the question whether quantitative studies accurately reflect the perspectives of QoL in HD should be explored further. As the QoL literature seems to be dominated by quantitative studies [7], future research on QoL should include more qualitative work, especially given the subjective nature of QoL and the value of qualitative studies in complementing and enriching quantitative research. Views of health care providers and caregivers on HDGECs’ QoL and how they differ from those of HDGECs themselves are welcome, especially in the later stages of HD, providing that researchers are aware of certain phenomena, such as the so-called response shift. Response shift and related concepts, such as the disability paradox and affective forecasting, should be investigated further, as they might play an important role throughout the course of the disease.

## Conclusions

The current study has built a first understanding of how QoL is defined from the perspectives of HDGECs and highlights a difference and adaptive shift in focus between premanifest and manifest HDGECs when defining their QoL. Premanifest HDGECs focused on an ideal life and redirected any HD-related concerns towards the future, whereas manifest HDGECs seem to focus on their actual reality by coming to terms with HD. Given these differences, we emphasize the importance of asking HDGECs about their own experiences when it comes to QoL, thereby taking into account the possible complexity of such questions for manifest HDGECs. Even though the management of symptoms and maintaining acceptable QoL are the ultimate goals in HD care [11, 12, 60], effective interventions targeting QoL are limited and further research is needed [7]. Our findings suggest that HDGECs may benefit from being informed about their future disease trajectory and capacity to adapt to deteriorations in functioning. Health care providers, in particular counsellors and social workers, can play an important role in informing HDGECs and caregivers about forming helpful beliefs about future adaptability and resilience, as well as in developing adaptive coping strategies. This might help HDGECs to manage their emotions towards the future and to adapt positively to their continuously changing realities and the transition into the diagnosed life phase. Techniques from positive psychology or acceptance and commitment therapy can assist with identifying (changes in) meanings and values in life to foster positive emotions and cognitions towards current and future experiences, as well as identify factors that contribute to a meaningful life [61, 62]. Peers might also have an important role to play in this, for instance by sharing their personal story. In providing optimal care, it is recommended to consider the individual as part of a complex, continuously changing environment, thereby taking into account their individual QoL experiences and tailor treatment and support accordingly.

## Data Availability

The data supporting the findings of this study are included in Table 3.

## Abbreviations

QoL: Quality of life
HRQoL: Health-related quality of life
HD: Huntington’s disease
HTT: Huntingtin gene
HDGECs: Huntington’s disease gene expansion carriers
COREQ: Consolidated criteria for reporting qualitative research
WHO: World health organization
